# Biographical

**Published:** 1897-05

**Authors:** 


### EDITORIAL.

### Biographical.

The following biography and memorial of Dr. H. W. Ray, of St. Joseph, Mich., was prepared by a committee of the Dental Society of South-western Michigan and ordered spread upon the records of that society and published in the Dental Journals:

Since our last meeting, one of our members, Dr. Hiram W. Ray, has been called to that beautiful home beyond, who departed this life March 17th, 1897, at 12:20 p. m. It has been said, that life is not measured by years, but by intensity. Scarcely fifty years from his birth, Dr. Ray passed to his final rest, but though called thence, in the prime of his useful vigorous manhood, he had accomplished much for the good of his fellow-men and if existence were counted by deeds, and not by years, he might well be called a venerable man.

For a long time he was closely connected with the best interests of St. Joseph, and was one of the prominent dentists of the state of Michigan. Both in professional and social circles, he occupied a position of influence and his reputation as a skilled dentist was by no means limited to St. Joseph. Though he resided in Michigan for many years, he was not a native of this state, but was born in LaFayette County, Wisconsin, in the city of Darlington, March 4th, 1847, being one of the eight children of John and Lucy Ray. The subject of this sketch, passed his boyhood years at Darlington, and in that vacinity until he completed his course in the common schools, after which he entered college and prosecuted his studies there for some time.

In 1865, on the 6th of December, he entered the office of Dr. James Brown, of Galena, Ills., where he gained his rudimentary knowledge of his profession; later, he entered the Philadelphia Dental College, and remained in that institution one year.

Opening an office at Bellevue, Ia., he conducted a general practice for three years, meantime he married Miss Isabella Wynkoop.

In November, 1875, he located at St. Joseph and there continued his practice until his death. Dr. Ray was a republican in his political preference and has occupied several positions of trust. Socially, he was a member of Occidental Lodge No. 56, F. & A. M., Calvin Brittain Chapter, No. 52, Malta Commandery No. 44, K. T., of Benton Harbor, Independent Order of Odd Fellows, K. O. T. M., and M. W.

Dr. Ray leaves a wife and three children, William H., Kittie Bell and Lawrence F., who continues his father's practice.

Dr. Ray was also a member of the Michigan Dental Association and Dental Society of South-western Michigan and was recognized as among the prominent men of his profession.

E. J. Backus,
H. C. Rockwell,
S. M. White,
*Committee.*

